# Influence of ruminal degradable intake protein restriction on characteristics of digestion and growth performance of feedlot cattle during the late finishing phase

**DOI:** 10.1186/2055-0391-56-14

**Published:** 2014-08-13

**Authors:** Dixie May, Jose F Calderon, Victor M Gonzalez, Martin Montano, Alejandro Plascencia, Jaime Salinas-Chavira, Noemi Torrentera, Richard A Zinn

**Affiliations:** Instituto de Investigaciones en Ciencias Veterinarias, UABC, Mexicali, Baja California 21100 México; Facultad de Medicina Veterinaria y Zootecnia, UAT, Cd. Victoria, Tamaulipas, 87000 México; Department of Animal Science, University of California, Davis E. Holton Rd, El Centro, CA 92242 USA

**Keywords:** Cattle, Degradable protein, Digestion, Growth performance

## Abstract

Two trials were conducted to evaluate the influence of supplemental urea withdrawal on characteristics of digestion (Trial 1) and growth performance (Trial 2) of feedlot cattle during the last 40 days on feed. Treatments consisted of a steam-flaked corn-based finishing diet supplemented with urea to provide urea fermentation potential (UFP) of 0, 0.6, and 1.2%. In Trial 1, six Holstein steers (160 ± 10 kg) with cannulas in the rumen and proximal duodenum were used in a replicated 3 × 3 Latin square experiment. Decreasing supplemental urea decreased (linear effect, *P* ≤ 0.05) ruminal OM digestion. This effect was mediated by decreases (linear effect, *P* ≤ 0.05) in ruminal digestibility of NDF and N. Passage of non-ammonia and microbial N (MN) to the small intestine decreased (linear effect, *P* = 0.04) with decreasing dietary urea level. Total tract digestion of OM (linear effect, *P* = 0.06), NDF (linear effect, *P* = 0.07), N (linear effect, *P* = 0.04) and dietary DE (linear effect, *P* = 0.05) decreased with decreasing urea level. Treatment effects on total tract starch digestion, although numerically small, likewise tended (linear effect, *P* = 0.11) to decrease with decreasing urea level. Decreased fiber digestion accounted for 51% of the variation in OM digestion. Ruminal pH was not affected by treatments averaging 5.82. Decreasing urea level decreased (linear effect, *P* ≤ 0.05) ruminal N-NH and blood urea nitrogen. In Trial 2, 90 crossbred steers (468 kg ± 8), were used in a 40 d feeding trial (5 steers/pen, 6 pens/ treatment) to evaluate treatment effects on final-phase growth performance. Decreasing urea level did not affect DMI, but decreased (linear effect, *P* ≤ 0.03) ADG, gain efficiency, and dietary NE. It is concluded that in addition to effects on metabolizable amino acid flow to the small intestine, depriving cattle of otherwise ruminally degradable N (RDP) during the late finishing phase may negatively impact site and extent of digestion of OM, depressing ADG, gain efficiency, and dietary NE.

## Background

Because of its low cost per unit of N compared with most sources of natural protein, urea is a primary source of supplemental N in conventional steam-flaked corn-based finishing diets for feedlot cattle [1]. In a review of nutrition consultant recommendations across 11 states in the USA, Vasconcelos and Galyean [2] observed that on average, flaked corn-based finishing diets contained 13.5% CP with 1.2% of supplemental urea (approximately 64% DIP). Although dietary formulation in this manner is expected to meet urea fermentation potential (UFP) for optimal microbial growth, it may exceed protein requirements for cattle growth, particularly during the late finishing phase. Preston [3] proposed the feasibility of restricting protein supplementation during the late finishing phase as a means of minimizing N excess and associated environmental impact [1, 4] without detrimentally affecting cattle performance. However, the impact of this practice on digestive function and cattle growth-performance has received limited research attention. The aim of this study was to evaluate the influence of UFP for optimal microbial growth on characteristics of digestion and growth performance of feedlot cattle during the late finishing phase.

## Methods

All procedures involving animal care and management were in accordance with and approved by the University of California, Davis, Animal Use and Care Committee.

### Trial 1

Six Holstein steers (160 ± 10 kg) with cannulas in the rumen and proximal duodenum [5] were used in 3 × 3 replicated Latin square experiment. Burroughs *et al.* [6] proposed that amount of degradable intake protein (DIP) necessary to optimize microbial growth was equivalent to the net microbial protein synthesis. Accordingly, the urea fermentation potential of the diet (percentage of additional urea that may be added to the diet in order to optimize microbial growth) would be equivalent to: (0.104TDN- DPI)/2.8, where TDN is expressed as a percentage, and DPI is expressed as the percentage of RDP in the basal diet before urea supplementation. Accordingly, treatments consisted of a steam flaked corn-based finishing diet adjusted for restriction of rumen DIP to provide urea fermentation potentials of 0 (UFP-0), 0.6 (UFP-0.6) and 1.2% (UFP-1.2). Composition of experimental diets is shown in Table 1. Chromic oxide (0.40%, DM basis) was included in diets as a digesta marker. Dry matter intake was restricted to 4.0 kg/d (2.2% of BW daily), and feed was offered in equal portions at 0800 and 2000 daily. The three experimental periods consisted of a 10-d diet adjustment period followed by a 4-d collection period. During the collection period duodenal and fecal samples were taken from all steers, twice daily as follows: d 1, 1050 and 1450; d 2, 0900 and 1500; d 3, 0730 and 1330, and d 4, 0600 and 1200. Individual samples consisted of approximately 750 mL of duodenal chyme and 200 g (wet basis) of fecal material. Samples from each steer and within each collection period were composited for analysis. During the final day of each collection period, 4 h after feeding, ruminal and blood samples were collected from each steer via ruminal cannula and caudal venous respectively. Ruminal fluid pH was determined by inserting a pH electrode into the freshly collected samples. The ruminal fluid sample was divided into two parts: 40 mL was measured into a plastic bag, placed in an ice bath, and carried to a laboratory for determination of N-NH in fresh ruminal fluid [7]. The remainder was strained through four layers of cheesecloth. Ten mL of freshly prepared 25% (wt/vol) metaphosphoric acid was added to 40 mL of strained ruminal fluid, 10 mL were then centrifuged (17,000 × *g* for 10 min), and supernatant fluid was stored at −20°C for VFA analysis. Upon completion of the trial, ruminal fluid was obtained via the ruminal cannula from all steers and composited for microbial isolation via differential centrifugation [8]. The microbial isolates were prepared for analysis by oven drying at 70°C and then grinding with mortar and pestle. Feed, duodenal, and fecal samples were prepared for analysis by oven drying at 70°C and then grinding in a laboratory mill. Samples were then oven dried at 105°C until no further weight loss occurred and stored in tightly sealed glass jars. Samples were subjected to all or part of the following analysis: DM (oven-drying at 105°C until no further weight loss), ash, N-NH, Kjeldahl N [9], NDF-adjusted for insoluble ash [10], purines [11], starch [12] and VFA concentrations of ruminal fluid (gas chromatography; [13]), GE (adiabatic bomb calorimetry), and chromic oxide [14]. Duodenal flow and fecal excretion of DM were calculated based on marker ratio, using chromic oxide. Microbial organic matter (MOM) and microbial N (MN) leaving the abomasum were calculated using purines as a microbial marker [11]. Organic matter fermented in the rumen was considered equal to OM intake minus the difference between the amount of total OM reaching the duodenum and MOM reaching the duodenum. Feed N escape to the small intestine was considered equal to total N leaving the abomasum minus N-NH, microbial, and endogenous N (0.195 g/kg W^0.75^; [15]). Methane production (mol/mol of glucose equivalent fermented) was estimated based on the theoretical fermentation balance for observed molar distribution of VFA [16]. Whole blood samples were centrifuged and the plasma frozen for BUN analysis. The blood samples collected were centrifuged and the plasma analyzed for Blood Urea Nitrogen (BUN) by slide method using Vitros Bun/Urea DT60 II (Ortho Clinical Diagnostics, Inc., Rochester, NY), and ruminal N-NH [7]. The effects of the urea level on characteristics of digestion in cattle were analyzed as a 3 × 3 replicated Latin square design using the MIXED procedure (SAS Inst. Inc., Cary, NC). The fixed effect consisted of treatment, and random effects consisted of steer and period. The statistical model for the trial was as follows:

**Table 1:** **Diet composition of experiment 1 and 2** ^**1**^

	Urea fermentation potential		
Item	0	0.6	1.2
Ingredient (g/kg of DM)			
Steam flaked corn	797.5	803.0	809.0
Sudangrass hay	50.0	50.0	50.0
Alfalfa hay	50.0	50.0	50.0
Urea	12.5	7.0	1.0
Cane molasses	50.0	50.0	50.0
Yellow grease	20.0	20.0	20.0
Limestone	14.0	14.0	14.0
Trace mineral salt^2^	4.0	4.0	4.0
Magnesium oxide	2.0	2.0	2.0
Monensin^3^	0.022	0.022	0.022
Nutrient composition (DM basis)^4^			
NE_m_ (Mcal/kg)	2.23	2.24	2.25
NE_g_ (Mcal/kg)	1.56	1.56	1.58
DE (Mcal/kg)	3.86	3.86	3.89
CP (g/kg)	130.0	115.0	99.1
RDP (g/kg of CP)	648	600	530
NDF (g/kg)	125.0	125.0	125.0
Calcium (g/kg)	6.6	6.6	6.6
Phosphorus (g/kg)	2.8	2.8	2.8

^1^Chromic oxide (0.40%) was added in substitution of corn grain as a digesta marker in Trial 1. RDIP, rumen degradable intake protein. UFP, estimated urea fermentation potential.

^2^Trace mineral salt contained: CoSO4, 0.068%; CuSO4, 1.04%; FeSO4, 3.57%; ZnO, 1.24%; MnSO4, 1.07%; KI, 0.052%; and NaCl, 92.96%.

^3^Rumensin80 (Elanco Animal Health, Greenfield, IN).

^4^Based on tabular values for individual feed ingredients (NRC, [17]).



where: Y_ijk_ is the response variable, μ is the common experimental effect, R_l_ is the replicated effect, S_i_ is the steer effect within replicate, P_j_ is the period effect within replicate, T_k_ is the treatment effect and E_ijk_ is the residual error. Treatment effects were tested using the following contrasts: 1) linear effect of the urea level, and 2) quadratic effect of the urea level, which were determined according to SAS (SAS Inst., Inc., Cary, NC; Version 9.1).

### Trial 2

Ninety crossbred steers with an average initial weight of 468 ± 8 kg were used in a 40 d finishing trial to evaluate the treatment effects on growth performance. Steers had a purchase weight of 214 ± 14 kg and had been on feed 197 d before initiation of the study. Steers had been implanted with Synovex-S (Zoetis, Florham Park, NJ) upon arrival into the feedlot and with Revalor-S (Merck Animal Health, Summit, NJ) on d 98. Ten d prior to initiation of the study steers were weighed, reimplanted with Revalor-S, blocked by weight and randomly allotted within weight groupings to 18 pens (5 steers/pen). Pens were 43 m^2^, with 22 m^2^ of overhead shade, automatic waterers, and 2.4 m long fence-line feed bunks. Dietary treatments were the same as those used in Experiment 1. All steers received the UFP-0 diet for 10 d prior to initiation of the trial. Diets were prepared at weekly intervals and stored in plywood boxes located in front of each pen. Steers were allowed free access to dietary treatments. Fresh feed was provided twice daily. Individual steers were weighed upon initiation and completion of the trial. In the calculation of steer performance live weights were reduce 4% to adjust for digestive tract fill. Estimates of steer performance were based on pen means. Net energy values for each diet were calculated from estimates of energy gain (EG, Mcal/d) based on growth-performance; EG = 0.0557 BW^0.75^ (ADG^1.097^), where EG is the daily energy deposited (Mcal/d), BW is the mean shrunk body weight (full weight × 0.96) and maintenance energy expended (EM, Mcal/d); EM = 0.077 BW^0.75^ [18]. Dietary NEg was derived from NE_m_ by the equation: NE_g_ = 0.877 NE_m_ - 0.41 [19]. Dry matter intake is related to energy requirements and dietary NE_m_ according to the equation: DMI = EG / NE_g_), and can be resolved for estimation of dietary NE by means of the quadratic formula: , where x = NE_m_, a = -0.877 DMI, b = 0.877 EM + 0.41 DMI + EG, and c = -0.41 EM [19].

All steers were harvested on the same day. Each carcass was weighed at time of slaughter to determine dressing percentage [20]. Performance (gain, gain efficiency, and dietary energetics) and carcass data were analyzed as a randomized complete block design; the experimental unit was the pen. The MIXED procedure of SAS [21] was used to analyze the variables. The fixed effect consisted of treatment, and pen was the random component. Treatments effects were tested using the following contrasts: 1) linear effect of the urea level, and 2) quadratic effect of the urea level, which were determined according to SAS [21].

## Results and discussion

The influence of dietary treatments on ruminal and total tract digestion is shown in Table 2. Decreasing supplemental urea decreased (linear effect, *P* ≤ 0.05) ruminal OM digestion. This effect was mediated by decreases in ruminal digestibility of NDF (linear effect, *P* = 0.05), starch (linear effect, *P* = 0.09) and N (linear effect, *P* = 0.04). Likewise, Zinn *et al.* [22] observed decreased ruminal digestion of OM, NDF and starch in response to decreasing urea supplementation of a steam-flaked corn-based finishing diet fed to feedlot steers [22].

**Table 2:** **Influence of dietary treatments on characteristics of digestion**

	Urea fermentation potential			*P -*value		
Item	0	0.6	1.2	Linear	Quadratic	SEM
Steer replications	6	6	6			
Intake (g/d)						
DM	3556	3553	3551			
OM	3343	3359	3378			
NDF	453	455	457			
N	66.2	57.6	48.2			
Starch	1907	1919	1932			
GE (Mcal/d)	15.2	15.2	15.3			
Flow to the duodenum (g/d)						
OM	1655	1749	1894	0.05	0.76	83.0
NDF	338	370	456	0.05	0.55	42.0
Starch	385	441	533	0.08	0.77	61.0
Total N	76.4	68.8	64.4	0.04	0.70	4.0
Microbial N	40.6	34.7	31.4	0.04	0.69	3.0
NH-N	2.30	2.04	1.58	0.06	0.72	0.3
Non-ammonia N	74.1	66.7	62.9	0.04	0.66	3.8
Feed N	24.7	23.2	22.7	0.20	0.71	0.9
Ruminal digestibility, %						
OM	62.6	58.3	53.23	0.04	0.91	0.3
NDF	25.3	18.6	0.30	0.05	0.54	0.9
Starch	79.8	77.0	72.4	0.09	0.78	0.3
Feed N	62.6	59.7	52.9	0.04	0.64	0.5
Microbial efficiency^1^	19.4	17.8	17.5	0.06	0.40	0.7
N efficiency^2^	1.12	1.16	1.30	0.05	0.44	0.07
Fecal excretion (g/d)						
OM	624	705	756	0.06	0.77	48.0
NDF	282	319	348	0.06	0.87	25.0
Starch	35.9	49.0	56.6	0.10	0.78	9.4
Total N	20.9	22.2	21.8	0.48	0.44	1.0
GE (Mcal/d)	3.28	3.66	3.90	0.05	0.75	0.84
Postruminal digestibility (% of flow to duodenum)						
OM	62.2	59.7	60.0	0.37	0.49	1.9
NDF	15.3	11.2	22.8	0.41	0.33	7.3
Starch	90.5	89.1	89.3	0.60	0.69	1.9
Total N	72.6	67.6	66.2	0.08	0.53	2.7
Total tract digestibility (% of intake)						
OM	81.3	79.0	77.6	0.06	0.75	1.4
NDF	37.8	29.8	23.9	0.07	0.85	5.3
Starch	98.1	97.4	97.1	0.11	0.77	0.5
Total N	68.5	61.5	54.9	0.04	0.97	4.5
DE, %	78.5	76.0	74.5	0.05	0.74	1.4
DE, Mcal/kg	3.36	3.26	3.20	0.05	0.73	0.06

^1^Microbial N, g/kg OM fermented.

^2^Nonammonia N flow to the small intestine as a fraction of N intake.

Passage of non-ammonia N to the small intestine decreased (linear effect, *P* = 0.04) with decreasing dietary urea level. This effect was due to decreased (linear effect, *P* = 0.04) MN synthesis. Taking into consideration energy intake alone, predicted flow of MN to the small intestine was 48g/d ([17], Level 1). Accordingly, with decreasing urea level, the observed flow of MN to the small intestine was 85, 73, and 65% of predicted flow for UFP-0, UFP-0.6, and UFP-1.2, respectively. This decline in net synthesis is consistent with [19] who observed that MN flow to the small intestine declines with decreasing DIP below 100 g/kg of total tract digestible OM. For the present study, DIP averaged 95, 81, and 61g/kg total tract digestible OM for UFP-0, UFP-0.6, and UFP-1.2, respectively. Thus, it is apparent that as DIP intake drops below 95 g/kg digestible OM there is not sufficient compensation in ruminal N recycling to maintain microbial growth, and as microbial growth declines, likewise, ruminal OM digestion declines.

There were no treatment effects (*P* = 0.20) on passage of feed N to the small intestine. Notwithstanding decreased non-ammonia N flow to the small intestine with decreasing urea level, ruminal N efficiency (non-ammonia N flow to the small intestine as a fraction of N intake) increased (linear *P* < 0.05), reflecting increased contribution of recycled N into microbial protein synthesis, consistent with the observation that ruminal N flux increases inversely with dietary N concentration [23]. Observed DIP (Table 2) averaged 103% of expected based on tabular values ([17]; Table 1) for the three dietary treatments.

Total tract digestion of OM (linear effect, *P* = 0.06), NDF (linear effect, *P* = 0.07), N (linear effect, *P* = 0.04) and dietary DE (linear effect, *P* = 0.05) decreased with decreasing urea level. Treatment effects on total tract starch digestion, although numerically small, likewise tended (linear effect, *P* = 0.11) to decrease with decreasing urea level. Decreased fiber digestion accounted for 51% of the variation in OM digestion. In a previous study involving steam-flaked corn-based finishing diets in which urea was the sole source of supplemental N [22], increasing urea level from 1.0 to 1.6% of the steam-flaked corn in the diet (an upper level similar to that of the present study; Table 1) likewise enhanced total tract OM and fiber digestion. In contrast Zinn and Shen [19] observed removal of urea from a steam-flaked corn-based growing-finishing diet markedly depressed ruminal OM digestion and flow of MN to the small intestine but did not affect total tract OM digestion. Treatment effects on apparent N digestion were largely a function of the N content of the diet brought about by changes in dietary urea level [24].

Treatment effects on ruminal pH, VFA molar proportions, and BUN are showen in Table 3. Ruminal pH (measured 4-h postprandium) was not affected (*P* = 0.51) by treatments, averaging 5.82. Upon hydrolysis, dietary urea can have an appreciable alkalizing effect on ruminal pH during the first hour post-feeding [25]. However by 4 h postprandium, the effect of urea supplementation of corn-based diets on ruminal pH has been negligible [22, 26, 27].

**Table 3:** **Treatment effects on ruminal pH, VFA molar proportions and BUN**

	Urea fermentation potential			*P –*value		
Item	0	0.6	1.2	Linear	Quadratic	SEM
Ruminal pH	5.75	5.86	5.84	0.51	0.59	0.10
Ruminal N-NH (mg/dL)	5.37	4.69	3.89	0.05	0.91	0.56
Total VFA (m*M*)	95.9	105	94.2	0.83	0.21	5.4
Ruminal VFA (mol/100 mol)						
Acetate	46.8	49.2	57.5	0.08	0.54	4.6
Propionate	36.1	30.1	20.3	0.04	0.74	5.7
Isobutyrate	1.19	1.17	0.89	0.30	0.60	0.23
Butyrate	12.0	15.1	17.7	0.17	0.93	3.1
Isovalerate	1.53	1.77	0.83	0.19	0.20	0.41
Valerate	2.36	2.63	2.83	0.42	0.95	0.48
Acetate:propionate	1.34	1.85	2.94	0.05	0.63	0.59
Methane^1^	0.35	0.44	0.60	0.04	0.70	0.09
BUN (mg/dL)	4.43	2.80	1.45	<0.01	0.53	0.25

^1^Methane production (mol/mol of glucose equivalent fermented) was estimated based on the theoretical fermentation balance for observed molar distribution of VFA [16].

Decreasing urea level decreased (linear effect, *P* < 0.01) ruminal N-NH. The N-NH concentration has been reported to increase immediately after feeding for 2 to 3 h [28, 29]. Satter and Roffler [30] observed a close relationship (R^2^ = 0.92) between the level of dietary CP and ruminal N-NH concentration at given dietary TDN. Likewise, in the present study dietary CP explained 88% of the variation ruminal N-NH concentration. Blood urea nitrogen (BUN) concentration 4 h postprandium also decreased (linear effect, *P* < 0.01) with decreasing urea supplementation. Blood urea nitrogen is also closely associated dietary CP and ruminal N-NH concentrations [31, 32]. Consistent with Zinn *et al.* [22], decreasing urea level increased ruminal acetate:propionate molar ratio (linear effect, *P* = 0.05), and estimated methane production (mol/mol glucose equivalent fermented; linear effect, *P* = 0.04).

Treatment effects on growth performance of feedlot steers are shown in Table 4. Decreasing urea level did not affect DMI (*P* = 0.32), but decreased ADG (linear effect, *P* < 0.01), gain efficiency (linear effect, *P* < 0.01), and dietary NE (linear effect, *P* = 0.03). Few research has evaluated the influence of marked RDP restriction on growth-performance and dietary NE in feedlot cattle fed steam-flaked corn-based finishing diets. As with the present study, Zinn *et al.* [22] observed a linear increase in urea resulted in a linear increase in ADG, gain efficiency and dietary NE linearly increased. The UFP of the basal unsupplemented diet in that trial was 1.36%, indicating that cattle performance may be enhanced when level of urea supplementation exceeded that necessary for maximal ruminal microbial protein synthesis. As with steam-flaked corn, urea supplementation of dry rolled corn-based finishing diets to meet the UFP also enhanced ADG and gain efficiency [26, 33].

**Table 4:** **Treatment effects on growth performance and carcass weight of feedlot steers**

	Urea fermentation potential			*P-*value		
Item	0	0.6	1.2	Linear	Quadratic	SEM
Days on test	40	40	40			
Pen replicates	5	5	5			
Live weight (kg)^1^						
Initial	469	465	470	0.83	0.28	3.22
Final	510	502	501	0.17	0.54	4.40
DMI (kg/d)	7.32	6.99	6.94	0.32	0.67	0.26
ADG (kg/d)	1.04	0.93	0.78	<0.01	0.71	0.06
G:F	0.142	0.134	0.112	<0.01	0.32	0.005
Diet NE (Mcal/kg)						
Maintenance	2.37	2.33	2.18	0.03	0.37	0.21
Gain	1.67	1.64	1.50	0.03	0.37	0.21
Observed/expected NE						
Maintenance	1.07	1.05	0.98	0.03	0.37	0.02
Gain	1.09	1.07	0.98	0.03	0.37	0.03
HCW (kg)	336	331	330	0.16	0.67	3.02
Dressing (%)	65.9	66.0	65.8	0.80	0.67	0.23

^1^Initial and final weights were reduced 4% to adjust for digestive tract fill.

The decrease in dietary NE due to restriction of rumen degradable intake protein observed in the growth performance trial (Table 4) is consistent with the decrease in dietary DE observed in the metabolism trial (Table 2). However, why cattle didn’t simply compensate for this difference in NE by increasing energy intake to maintain their growth potential is puzzling. A comparison of requirements and estimated supply of metabolizable protein and the amino acids methionine and lysine for the various dietary treatments is given in Table 5. As per NRC [17], metabolizable protein supply was estimated as 80% of undegraded intake crude protein plus microbial crude protein entering small intestine in Trial 1 (Table 2), adjusted for level of intake of steers in Trial 2 (Table 4). Metabolizable amino acid supply was based on diet composition (Table 1) and corresponding tabular amino acid composition of RUP for individual feed ingredients and average amino acid composition of ruminal bacteria [17]. Metabolizable protein and amino acid requirements were based on average body weight and daily weight gain (Trial 2; NRC, [17], Level 1). As expected, estimated metabolizable protein and amino acid supply decreased with increasing UFP. Across treatments, estimated metabolizable protein supply exceeded requirements by an average of 11%. Nevertheless, metabolizable protein supply for UFP-0.6 and UFP-1.2 were less (2 and 8%, respectively) than the estimated requirement to achieve daily weight gain observed with UFP-0 treatment. Particularly notable is the very close association between metabolizable methionine and lysine and requirements versus supply, indicative that daily weight gain may have been closely mediated by supply of these two amino acids. As corn (the major contributor of protein to the basal diet) is a particularly poor source of lysine, and methionine, the diminution of microbial protein synthesis brought about by restriction in RDP, was sufficient to restrict growth.

**Table 5:** **Treatment effects on metabolizable protein and amino acid supply** ^**1**^ **versus requirements** ^**2**^

	Urea fermentation potential		
Item	0	0.6	1.2
Metabolizable protein, g/d			
Supply	688	600	565
Requirement	613	574	493
Metabolizable methionine, g/d			
Supply	12.4	10.6	9.9
Requirement	12.3	11.5	9.9
Metabolizable lysine, g/d			
Supply	39.2	32.9	30.1
Requirement	39.3	36.7	31.5

^1^Metabolizable protein supply estimated as 80% undegraded intake crude protein and microbial crude protein entering small intestine (Trial 1), adjusted for level of intake. Metabolizable amino acid supply based on diet composition and corresponding tabular amino acid composition of undegradable intake protein for individual feed ingredients and average amino acid composition of ruminal bacteria (NRC, [17]).

^2^Metabolizable protein and amino acid requirements based on average body weight and daily weight gain (Trial 2; NRC, [17], Level 1).

## Conclusion

It is concluded that in addition to effects on net protein flow to the small intestine, depriving cattle of otherwise RDP during the late finishing phase may negatively impact site and extent of OM digestion, depressing ADG, gain efficiency, and dietary NE.
